# Personal factors associated with carpal tunnel syndrome (CTS): a case-control study

**DOI:** 10.1186/s12891-021-04941-y

**Published:** 2021-12-20

**Authors:** Eman Al Shahrani, Abeer Al Shahrani, Nassr Al-Maflehi

**Affiliations:** 1 Department of Family & Community Medicine, King Saud University Medical City, Riyadh, Saudi Arabia; 2 Department of Clinical Sciences, College of Medicine, Princess Nourah bint Abdulrahman University, Riyadh, Saudi Arabia; 3 Department of Periodontics & Community Dentistry, College of Dentistry, King Saud University, Riyadh, Saudi Arabia

**Keywords:** Carpal tunnel syndrome, CTS, Median nerve, Sociodemographic factors

## Abstract

**Background:**

Carpal tunnel syndrome (CTS) is one of the most common nerve entrapments in the upper limb. In Saudi Arabia, few studies have investigated CTS in the general population. This study aimed to determine the association between personal factors and CTS.

**Methods:**

A case-control study involved adults aged 18 and above. Cases were recruited from electrophysiology lab records as consecutive case series, while controls were individuals who were free of CTS symptoms according to the Boston Carpal Tunnel Questionnaire (BCTQ). The electronic medical records of participants were reviewed to obtain age, height, weight, medical conditions, and mobile numbers. Cases and controls were contacted via phone to complete a questionnaire that was designed based on previous literature. We used multivariate binary logistic regression to identify the personal factors significantly associated with CTS.

**Results:**

A total of 95 cases and 190 controls were included. Most of the participants were female (84.2%) and Saudi (93%). Most of cases were above 45 years of age (73.7%), while 84.7% were 45 year – old or younger among the control group. Stratified logistic regression showed that performance of household chores was significantly associated with CTS. While physical exercise associated with decreased odds of CTS.

**Conclusions:**

This study adds to the body of evidence on personal factors associated with CTS. However, the degree of differences in the age structure of the cases compared with the controls suggest that there is a considerable potential for residual confounding affecting the results.

**Trial registration number:**

Not applicable.

## Background

Carpal tunnel syndrome (CTS) is defined as “a symptomatic compression neuropathy of the median nerve at the level of the wrist” [1]. CTS is one of the most common entrapment neuropathies affecting the upper limb and carries a significant impact on work and daily activities [2]. It is characterized by the presence of sensory symptoms in the form of paraesthesia, dysaesthesia, with or without pain with subsequent loss of sensation and weakness affecting area innervated by median nerve [3].

Its prevalence varies widely in the literature; it is believed that the overall prevalence of CTS is 8.0% in the general population [4], particularly adults between the ages of 40-60 years [5]. Moreover, females have a two- to three-fold increased risk compared to males, especially around the time of menopause [6, 7].

The pathophysiology of CTS remains a debatable issue, with many theories behind its development. However, three theories were consistently presented in the literature: compression from adjacent structures, elevated carpal tunnel pressure, and nerve ischaemia [8, 9]. Furthermore, CTS is multifactorial and often caused by multiple patient-specific, social, medical, occupational, and environmental risk factors [10]. Several studies have suggested CTS has a significant occupational relationship [11], especially with high-risk jobs that involve manual forceful exertion, repetitive hand movements, hand–arm transmitted vibration, and bending/twisting of the wrists [12, 13]. However, in the absence of high-risk occupations, other nonoccupational factors may predominate, for instance obesity, thyroid disease, diabetes mellitus (DM), pregnancy, renal failure, rheumatoid arthritis (RA), alcoholism, primary amyloidosis, and drug toxicity [14 – 15]. In some cases, two or more of these risk factors may coexist, placing the individual at higher risk of developing CTS [16, 17]. Moreover, several recent studies have found that psychosocial factors such as psychological problems, low job satisfaction and low socioeconomic status are inconsistently associated with CTS [18, 19].

Although few studies have been carried out on CTS in Saudi Arabia, only a few studies have investigated demographic and medical variables related to CTS in the general population [20, 21]. Others have explored the impact of occupation on CTS [22, 23]. However, most of these local studies were of cross-sectional design, which is considered a major limitation. Thus, this case-control study aimed to determine the association between CTS and personal risk factors related to sociodemographic, medical, and daily habits.

## Methods

### Study design, setting and participants

We conducted an unmatched case-control study at a single university teaching hospital in Riyadh, Saudi Arabia, from February 2019 – April 2020. In 2018, approximately 494,034 patients visited outpatient clinics, with an average of 190 patients confirmed with CTS as per the Neurophysiology lab registry.

Cases were recruited from electrophysiology lab records as consecutive case series. Controls were recruited from outpatient clinics with convenient sampling techniques. We targeted cases within a year of receiving a confirmed diagnosis of CTS using NCS. Recruitment of cases and controls started in April 2019 – April 2020, and during this time, every individual who met the inclusion criteria and agreed to participate was included until we reached our target sample size.

The inclusion criteria were as follows:

1. Adults 18-year-old and above
2. The presence of two or more of the following symptoms: (1) numbness and tingling in the area innervated by the median nerve; (2) night-time paraesthesia; (3) pain in the wrist area radiating to the shoulder; and (4) falling of objects from the hand because of lack of sensation [24]..
3. Confirmed diagnosis by positive NCS as suggested by diminished median nerve conduction values (below 50 m/s) and/or increased motor latency (above 4 m/s) [25].

The inclusion criteria for controls:

1. Adults 18-year-old and above
2. Free of CTS symptoms, as evidenced by scores on the Boston Carpal Tunnel Questionnaire (BCTQ) [26].

### Sample size

Using open Epi, the estimated sample size was 236 participants based on confidence level (95%), power (85%), expected prevalence of CTS among controls and cases 8 and 23.6%, respectively [27, 28], with a ratio of controls to cases 2:1. Therefore, the required sample size was 79 cases and 157 controls. However, data collection continued until we reached the estimated sample size plus 15% to compensate for any incomplete questionnaires.

### Data collection & study variables

The records of the patients who underwent NCS in the Electrophysiology Laboratory at King Khaled University Hospital (KKUH), were reviewed for the period of November 2018 to April 2019 to identify confirmed CTS cases who met the inclusion criteria. We used the disease-specific Boston Carpal Tunnel Questionnaire (BCTQ) to screen controls before their enrolment. We accepted those who scored 11 points (“no symptoms”) in symptom severity and/or 8 points (“normal”) in functional status [29]. The electronic files of participants were reviewed to obtain age, height, weight, medical conditions, and contact numbers. Both cases and controls completed a questionnaire designed based on previous literature via phone interview [26, 28]. This questionnaire was discussed with all data collectors to reach a consensus on how to ask/explain the questions to the study participants. A pilot study with 10 cases and 10 controls was conducted to determine face validity and estimate the time required for data collection.

The questionnaire addressed (1) sociodemographic characteristics: age, gender, nationality, marital status, education, employment status (employed and unemployed), dominant hand, (2) information related to medical factors including: (smoking, the presence of diabetes mellitus (DM), hypertension (HTN), hypothyroidism, rheumatoid arthritis (RA), acromegaly, fracture in the distal radius, and for female participants: parity, pregnancy, menopause, hormonal replacement therapy (HRT) and oral contraceptive pills (OCPs) use. Additionally, calculated body mass index (BMI) was interpreted as three categories: normal (BMI < 24 kg/m^2^), overweight (BMI = 25 – 29 kg/m^2^) and obese (≥ 30 kg/m^2^). (3) Regarding occupation, we inquired about the sector of the occupation (healthcare, education, banking, manufacturing etc.), and detailed information about exposure to biomechanical factors such as forceful movements, awkward posture, and repetitive movements of the wrist was assessed using yes/no questions. Moreover, participants who currently did not seek a job, student, housewife, or nonworking retiree were classified as unemployed. (4) Information related to daily habits, including physical exercise, sleep, household chores, and use of electronic devices (desktops, laptops, tablets, and video game consoles). (5) Additional data about CTS among cases, such as affected hand/s, symptoms and their duration, and type of treatment received.

Most of our variables were categorical, and they were collected using yes/no questions. However, we collected few quantitative variables, and they were converted into categories such as age, parity, frequency of physical exercise (regular, occasional, and never) frequency of household chores (regular, occasional, and never), duration of sleep, and duration of electronic devices use.

### Statistical analysis

Statistical analysis was performed using SPSS version 25.0 (IBM, Armonk, NY, USA). Descriptive statistics were calculated (frequencies, means, standard deviations) to describe the categorical and numerical variables. The chi-square test and Fisher’s exact test were used to quantify the association between categorical variables. We performed univariate analyses to identify factors associated with CTS and then those factors were used to construct multivariate binary logistic regression model. Furthermore, we have carried out age and gender specific regression analyses. The associations were reported as odds ratios (ORs) with 95% confidence intervals (CIs). P-values and 95% confidence intervals were used to report the statistical significance and precision of the results, respectively; p-values ≤0.05 were considered statistically significant.

## Results

Among 103 cases and 251 controls, only ninety-five (n = 95) cases and one hundred ninety controls (n = 190) agreed to participate in the study and completed the questionnaire. Table 1 shows the participants’ sociodemographic characteristics. Most of the participants in the two groups were female (84.2%) and Saudi (93%). 73. 7% of cases were above 45 years of age, while 84.7% were 45 year – old or younger among the control group. Regarding participants’ education, 58.9% of cases received minimal schooling, while 70% of controls received university education. Moreover, 81.2% and 55. 3% of female cases and controls were having two and more children.

**Table 1:** Sociodemographic characteristics of the study population

Variable	Level	Cases (n = 95)		Controls (n = 190)		p-value
		n	%	n	%	
Age	≤ 45 years	25	26.3	161	84.7	< 0.001*
	> 45 years	70	73.7	29	15.3	
Gender	Male	15	15.8	30	15.8	1.0
	Female	80	84.2	160	84.2	
Marital status	Unmarried	17	17.9	47	24.7	0.19
	Married	78	82.1	143	75.3	
Nationality	Saudi	89	93.7	177	93.2	0.87
	Non-Saudi	6	6.3	13	6.8	
Education	High school and less	56	58.9	57	30	< 0.001*
	University and above	39	41	133	70	
Employment status	Unemployed	70	73.7	120	63.2	0.08
	Employed	25	26.3	70	36.8	
Parity	0 - 1	15	18.8	71	44.7	< 0.001*
	2 +	65	81.2	89	55.3	
Dominant hand	Right	88	92.6	178	93.7	0.74
	Left	7	7.4	12	6.3	

^*^p-value < 0.05

Regarding participants’ occupation, only 26.3 and 36.8% were employed among cases and controls, respectively. A nonsignificant trend involved working in high-risk manual occupations such as manufacturing, construction, and retail businesses. We also obtained information about biomechanical factors: 58% of cases and 74% of controls performed repetitive hand movements such as keyboarding. Additionally, the results showed that 26.8 and 11.5% of cases and controls performed work that involved forceful or strenuous hand movements, respectively. Moreover, work regularly requiring an awkward posture of the wrist or hand was observed in 78 and 53.8% of cases and controls, respectively. However, work that exposes the hands to vibration from tools, impact shock or rebound was only found in 7.3 and 2.6% of cases and controls, respectively.

We explored other personal factors, such as medical conditions and daily habits, as shown in Table 2. Most of the study population was female, in which 29.5% of cases were menopausal at the time of diagnosis. Only 2% were pregnant at the time of diagnosis. Moreover, most of the sample was overweight/obese (BMI above 25 kg/m2 in both cases and controls. Diabetes mellitus (DM) and hypertension (HTN) were the most observed chronic medical conditions among the study population. Furthermore, we inquired about daily habits such as physical exercise, smoking, sleeping, and household chores. Both cases and controls were mainly nonsmokers, nonexercising and performing household chores regularly. Regarding electronic device use, laptops were used in 40.6% of the study population, followed by PCs in 27.4%, while tablets and videogame consoles were rarely used (16.8 and 4.2%, respectively). However, this was excluded from the regression analyses due to relatively small observations and difficulty assessing whether the exposure to electronic devices was as part of occupation or daily activity and/or both.

**Table 2:** Medical characteristics and daily habits of the study population

Variable	Level	Cases (n = 95)		Controls (n = 190)		p-value
		n	%	n	%	
Menopause	No	52	65	149	93.1	< 0.001*
	Yes	28	35	11	6.9	
Pregnancy	No	78	97.5	130	81.3	< 0.001*
	Yes	2	2.5	30	18.7	
Body mass index (BMI)	Normal	9	9.5	59	31	< 0.001*
	Overweight	26	27.4	50	26.3	
	Obese	60	63.2	81	42.6	
Medical conditions	Diabetes	44	46.3	22	11.6	< 0.001*
	HTN	37	38.9	18	9.5	< 0.001*
	Hypothyroidism	16	8.4	17	17.9	0.02*
	Rheumatoid arthritis	3	3.2	8	4.2	0.66
	Acromegaly	0	0	1	0.5	0.48
	Fractured distal radius	3	3.2	9	4.7	0.3
Smoking	Smoker	3	3.2	9	4.7	0.53
	Non-smoker	92	96.8	181	95.3	
Exercise	Never	54	56.8	76	40	0.02*
	Occasional	28	29.5	67	35.3	
	Regular	13	13.7	47	24.7	
Sleeping posture	Supine	8	8.4	27	14.2	0.19
	Left lateral	13	13.7	31	16.3	
	Right lateral	66	69.5	108	56.8	
	Prone	8	8.4	24	12.6	
Household chores	Never	17	17.9	31	16.3	0.008*
	Occasional	34	35.8	38	20	
	Regular	44	46.3	121	63.7	

^*^p-value < 0.05

Moreover, night-time paraesthesia was the most common CTS symptom in 55.8% of cases, and 75.8% had these symptoms for more than three years. Bilateral involvement was confirmed in 70.5% of cases. Furthermore, 39% received conservative treatment in terms of wrist splinting, physiotherapy, and corticosteroid injections, while 30.5% underwent surgical decompression.

Multivariate binary logistic regression model was constructed based on statistically significant associated factors with CTS. The following factors were included in the model: age, education, DM, HTN, hypothyroidism, BMI, physical exercise, and household chores. Considering that age is potentially related to many variables especially medical conditions, we have carried out age – specific stratified regression analysis with age of 45 years as cut-off level as shown in Table 3. It has been found that CTS was significantly associated with performing household chores regularly (OR = 11.9, 95% CI 1.58 - 89.65) among participants younger than 45 years. On the other hand, the odds of having CTS was less than 1 among participants performing physical exercise occasionally (OR = 0.11, 95% CI 0.02 - 0.7) among the same group. While rest of the factors showed nonsignificant associations with CTS. Furthermore, female specific stratified regression analyses on parity, menopause and pregnancy showed non statistically significant association with CTS.

**Table 3:** Age- stratified multivariate logistic regression for personal factors associated with CTS among the study population

Factor	Level	OR (95% CI) Age ≤ 45 years	P-value	OR (95% CI) Age > 45 years	P-value
Educational level (Ref: University & above)	School & less	1.54 (0.41- 5.72)	0.52	0.79 (0.18 - 3.38)	0.75
Parity * (Ref: 2 and more)	0 – 1	1.26 (0.35 - 4.53)	0.73	1.41 (0.18 - 11.04)	0.74
Pregnancy*		0.25 (.04 - 1.41)	0.12	–	–
Menopause*		–	–	0.38 (0.12 - 1.24)	0.11
Body mass index (Ref: Obese)	Normal	0.46 (0.12 - 1.80)	0.26	0.22 (0.04 - 1.41)	0.11
	Overweight	0.26 (0.6 - 1.22)	0.09	1.6 (0.44 - 5.7)	0.48
Medical conditions	Diabetes	2.06 (0.34 - 12.62)	0.43	1.35 (0.40 - 4.56)	0.63
	Hypertension	4.7 (0.65 - 34.2)	0.13	1.23 (0.33 - 4.48)	0.76
	Hypothyroidism	3.44 (0.66 - 17.8)	0.14	1.24 (0.32 - 4.75)	0.75
Exercise (Ref: Never)	Occasional	0.11 (0.02 - 0.7)	0.02**	1.45 (0.3 - 7.14)	0.65
	Regular	0.33 (0.09 - 1.23)	0.1	1.65 (0.45 - 6.14)	0.45
Household chores: (Ref: Never)	Occasional	1.76 (0.27 - 11.47)	0.55	1.7 (0.07 – 42)	0.76
	Regular	11.9 (1.58 - 89.65)	0.02**	3.31 (0.13 - 86.28)	0.47

* Female – specific stratified regression analysis

**p-value < 0.05

## Discussion

It is thought that CTS is multifactorial disease with a significant work-related component [10], however, the impact of nonoccupational factors should not be neglected [30]. The results of this study might add to the existing body of evidence on personal factors associated with CTS especially those related to medical conditions and daily habits. The mean age was greater in case than in control group (p < 0.001), considering that age is potentially related to many variables especially medical ones, we carried out stratified logistic regression aiming to overcome imbalance between the two groups and allowing proper comparison. Other alternative options may include conducting an age matched case-control study or selecting only subjects aged 40 – 65 years-old. However, those options were not feasible at the time of study conduction. The degree of differences in the age structure of the cases compared with the controls suggest that there is a considerable potential for residual confounding affecting the results.

Most of our study population were females. We have expected this finding because the literature has indicated an association between gender and carpal tunnel syndrome [7,31, 32]. Multiple proposed theories explain this association; one is the presence of differences in wrist anthropometrics between males and females [33, 34]. It is also suspected that sex hormones play a role in a higher incidence of CTS among females, especially around the time of menopause [35] and during pregnancy [36]. In our study, we inquired about parity, pregnancy, menopause, use of hormonal replacement therapy, and OCPs. However, female specific stratified logistic regression did not show these associations, probably due to the small sample size.

Common clinical conditions that have proposed to be associated with CTS include DM (both type I and II), obesity, hypertension, thyroid disease, and arthritis [37 – 39]. Our results showed that Obesity, DM, HTN and hypothyroidism were more prevalent among the case group with a statistically significant p-value. However, OR and 95% CI were not significant after stratifying age into two groups with 45 years as a cut-off level.

Among factors related to daily habits, household chores showed a significant association with CTS among cases aged less than 45 years. This finding has not been explored widely in the literature and is inconsistent. One cross-sectional study found that household chores were a probable attributable factor for CTS among women [40], while an older study did not confirm this association [41]. We think that our finding might reflect a true association between performing household chores and CTS, knowing that housewives accounted for 42% of the study population. Moreover, the odds of having CTS were significantly decreased in participants that occasionally performing physical exercise (once – three times / week). Such finding is supported by a case control study reporting that among injured workers, CTS was significantly associated with not engaging in a vigorous exercise and poorer physical health [42]. To the best of our knowledge, this is the first local published case-control study that involved the general population in Riyadh, Saudi Arabia. We were keen to obtain a representative sample of a sufficient size. Nevertheless, we are aware of the limitations of this study, including the unmatched case-control design, the convenient sampling technique and lack of proper screening tools for CTS among controls.

## Conclusions

CTS is one of the most common types of nerve entrapment in the upper limb. It has a significant impact on daily tasks and quality of life. A large body of evidence exists on CTS as an occupational disease with much debate regarding its pathophysiology and associated factors. Thus, awareness of potential personal factors associated with this condition can aid in early diagnosis and management. Performing household chores might be associated with CTS in younger adults. On the other hand, engaging in physical exercise associated with decreased odds of CTS in the same age group. However, the degree of differences in the age structure of the cases compared with the controls suggest that there is a considerable potential for residual confounding affecting the results. Despite a large body of research on risk factors for CTS, a cohesive set of personal risk factors has yet to emerge from large, prospective, population-based studies.

## Data Availability

Raw data were generated at [King Saud University Medical City, Riyadh, Saudi Arabia]. The data are not publicly available to avoid compromising the privacy of research participants. However, derived data supporting the findings of this study are available upon request and approval from the corresponding author.

## Abbreviations

BMI: Body mass index
CTS: Carpal tunnel syndrome
DM: Diabetes mellitus
HTN: Hypertension
NCS: Nerve conduction study
OCPs: Oral contraceptive pills
OR: Odds ratio
RA: Rheumatoid arthritis
